# Supplementary material for: Identifying and characterizing scene representations relevant for categorization behavior
Source: Imaging Neurosci (Camb). 2025 Jan 21;3:imag_a_00449. doi: 10.1162/imag_a_00449 (PMC12319821; doi:10.1162/imag_a_00449)
Supplement: Supplementary Material [file imag_a_00449-supp.pdf]

Supplementary Information

Control Analysis: Accuracy as a behavioral measure for brain-behavior correlations

To assess the robustness of the observed brain-behavior correlations, we repeated the correlations between behavior and neural distances in all three tasks, using accuracy as the behavioral measure instead of reaction time (RT). Since distance-accuracy correlations are expected to be positive, unlike the distance-RT correlations, we inverted the sign of the distance-accuracy correlations to facilitate direct comparison with the RT-based results.

As shown in Fig. S1, the results for man-made/natural and basic-level categorization closely mirrored the patterns observed when RTs were used as the behavioral metric. Significant correlations between neural distances and accuracy were found in EVC and LOC (all $p<0.023$), but not PPA (both $p>0.134$), for both categorization tasks. For the fixation task, we found significant correlations in LOC and PPA (both $p<0.028$), but not in EVC ($p=0.194$).

The searchlight results revealed significant positive correlations for the man-made/natural task at the border between occipital and ventral-temporal cortex as well as at the border between occipital and posterior parietal cortex. In addition, negative correlations were observed in the right occipital cortex only, consistent with the RT-based analyses. For the basic-level task, we found significant correlations in posterior and lateral parts of occipital cortex, analogous to the RT-based results. For the fixation task, we found no significant correlations ($p=0.099$), likely because of overall smaller effects compared with RTs as a behavioral metric.

In sum, while the results for accuracy were weaker and showed some minor differences for the fixation task, the overall pattern of results with accuracy as a behavioral metric were qualitatively similar to the ones with RTs. These findings indicate that the observed brain-behavior relationships are largely not specific to RTs but rather reflect a general link between neural representations and human behavior.

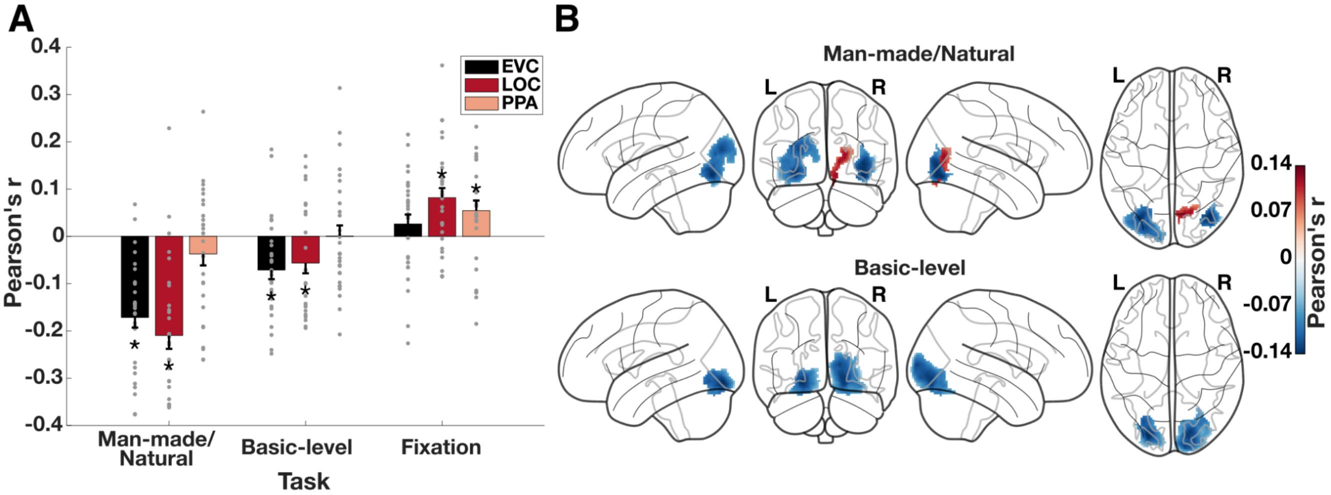

Supplementary Figure S1. Brain-behavior correlations using accuracy as the behavioral measure. A) Distance-accuracy correlations in EVC, LOC and PPA.

Significant correlations were found in EVC and LOC for the man-made/natural and basic-level categorization tasks, while no significant correlations were observed in PPA. For the fixation task, we found significant correlations in LOC and PPA, but not in EVC. Note that the distance-accuracy correlations were inverted to enable direct comparison with RT-based results. Error bars depict the standard error of the mean across participants. Stars above or below the bars indicate significant results ($p<.05$, FDR-corrected). **B) Distance-accuracy correlations across visual cortex.** Significant negative correlations were observed at the border between occipital and lateral occipital regions with positive correlations in occipital cortex for the man-made/natural task. For basic-level categorization, significant correlations were found in posterior and lateral parts of occipital cortex. These results are consistent with those obtained using RTs, demonstrating a robust relationship between neural representations and behavior across different tasks and behavioral measures.

Analysis of Additional Scene-Sensitive Regions (OPA and RSC)

To investigate the role of other scene-sensitive regions beyond PPA, we extended our analyses to include the occipital place area (OPA) and the retrosplenial cortex (RSC). Specifically, we evaluated decoding accuracies and distance-RT correlations for all three tasks across these regions, as well as in early visual cortex (EVC), lateral occipital complex (LOC), and parahippocampal place area (PPA) for comparison.

As shown in Fig. S2A, we observed significant decoding accuracies in OPA and RSC for both categorization tasks (all $p<0.001$), suggesting that these regions represent scene category information. This finding is consistent with their well documented role in scene processing (Epstein and Baker 2019). In addition, we assessed the relationship between neural representations and behavior by correlating neural distances with RTs in each region of interest (ROI) for each task separately. Fig. S2B shows the correlation between neural distances and RTs for both categorization tasks across ROIs. While significant negative correlations were found in EVC and LOC (all $p<0.003$), no significant correlations were observed for RSC or OPA for either task (all $p>0.253$). This suggests that while OPA and RSC represent scene categories, there is no positive evidence for a link to behavior as investigated here in these regions. Finally, we tested for interference between scene representations and an orthogonal task by assessing the correlation between neural distances for man-made/natural categorization and RTs in the fixation task. There were only significant positive correlations in EVC and LOC (both $p<0.004$) but not in any of the scene-selective regions including OPA and RSC (all $p>0.120$). These results further corroborate the absence of positive evidence for a link between scene representations in OPA and RSC with behavior. In sum, these analyses support our findings in PPA and extend them to a broader selection of scene-selective regions.

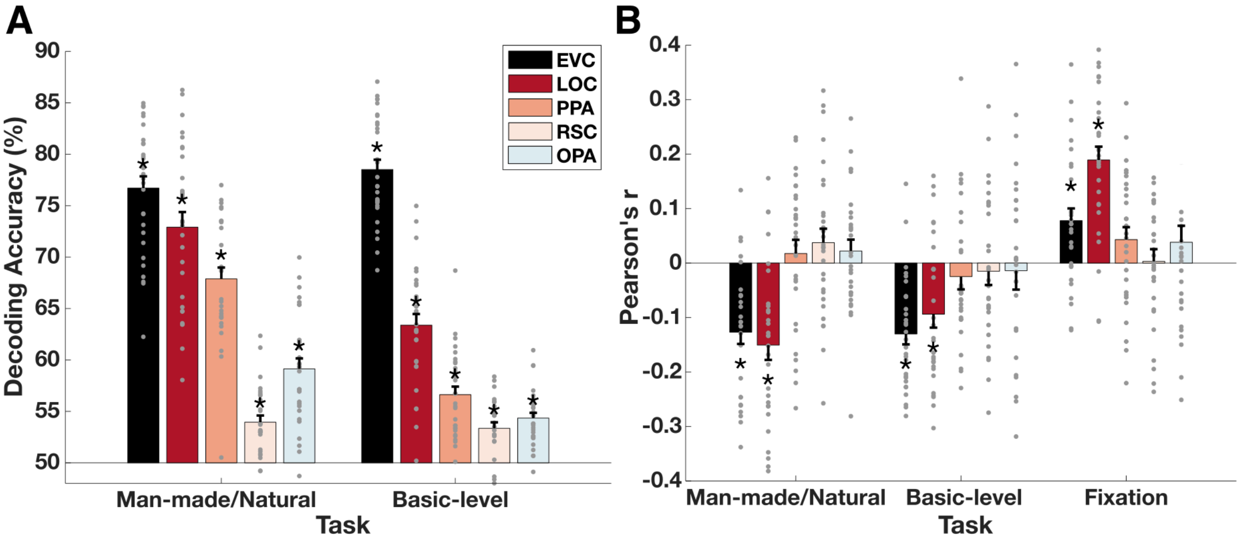

Supplementary Figure S2. Decoding accuracy and correlation between neural distances and reaction times (RTs) for extended ROIs. A) Scene category decoding. Significant decoding accuracies were observed in all ROIs for both man-made/natural and basic-level categorization. **B) Distance-RT correlations for all three tasks.** Significant distance-RT correlations for man-made/natural and basic-level categorization were only observed in EVC and LOC but not in any of the scene-selective regions (PPA, OPA, RSC). Analogous to the results for the categorization tasks, for the fixation task there were no significant distance-RT correlations in any of the scene-selective regions. Grey points indicate data points for individual subjects. Error bars depict the standard error of the mean across participants. Stars above or below the bars indicate significant results ($p<0.05$, FDR-corrected).

Control analysis: Randomly Initialized Networks

In order to determine whether our results based on neural network activations were indeed driven by visual feature representation learned during training the model on a scene categorization task, we performed a control analysis using randomly initialized networks. Specifically, we compared the shared variance between neural distances, behavioral RTs, and distances derived from randomly initialized and scene-trained networks.

For this analysis, we extracted activations from each layer of the randomly initialized networks and used these activations to train SVM classifiers for both man-made/natural and basic-level categorization. We then derived network distances by testing the classifiers on activations from the experimental images. Subsequently, we calculated the shared variance between these distances, the neural distances, and behavioral RTs for EVC and LOC separately. Finally, we computed the difference in explained variance between trained and untrained networks to quantify the effect of model training on the results.

As shown in Fig. S3, we found significant increases in explained variance for trained models compared to untrained models across most networks and layers and for both ROIs in the man-made/natural categorization task (all $p<0.043$). However,

for some layers there were no significant improvements, specifically: for EVC layers 1 and 6 in ResNet18, layer 5 in ResNet50, layer 1 in AlexNet, layer 2 in DenseNet161 (all $p>0.053$) and for LOC only in layer 1 in ResNet50 ($p=0.345$). Notably, a significant decrease was found in the first layer of DenseNet161 for man-made/natural categorization in LOC ($p=0.026$). For basic-level categorization, there were significant improvements in most layers except for the first and last layers in EVC. In LOC, the effects were less widespread, with significant increases primarily in the intermediate layers. Specifically, in EVC there were significant improvements in all layers except for layer 1 in ResNet18 and ResNet50, layer 6 for AlexNet, and layer 1 for DenseNet161 (all $p>0.182$). In LOC, there were significant improvements in all layers except for layers 1,5,6 in ResNet18 and ResNet50, and layer 6 in AlexNet and DenseNet161 (all $p>0.059$).

In sum, the trained models demonstrated significant improvements over the untrained models, with more restricted improvements for basic-level categorization in LOC. These findings suggest that model training is a crucial factor in explaining the shared variance between neural network activations, brain representations, and behavior. This is in line with the assumption that deep neural networks, when trained on object/scene categorization tasks, develop visual feature representations that closely match those in the human brain. Importantly, the results support our initial conclusion that particular visual features learned by the networks best explain the link between brain and behavior in scene categorization.

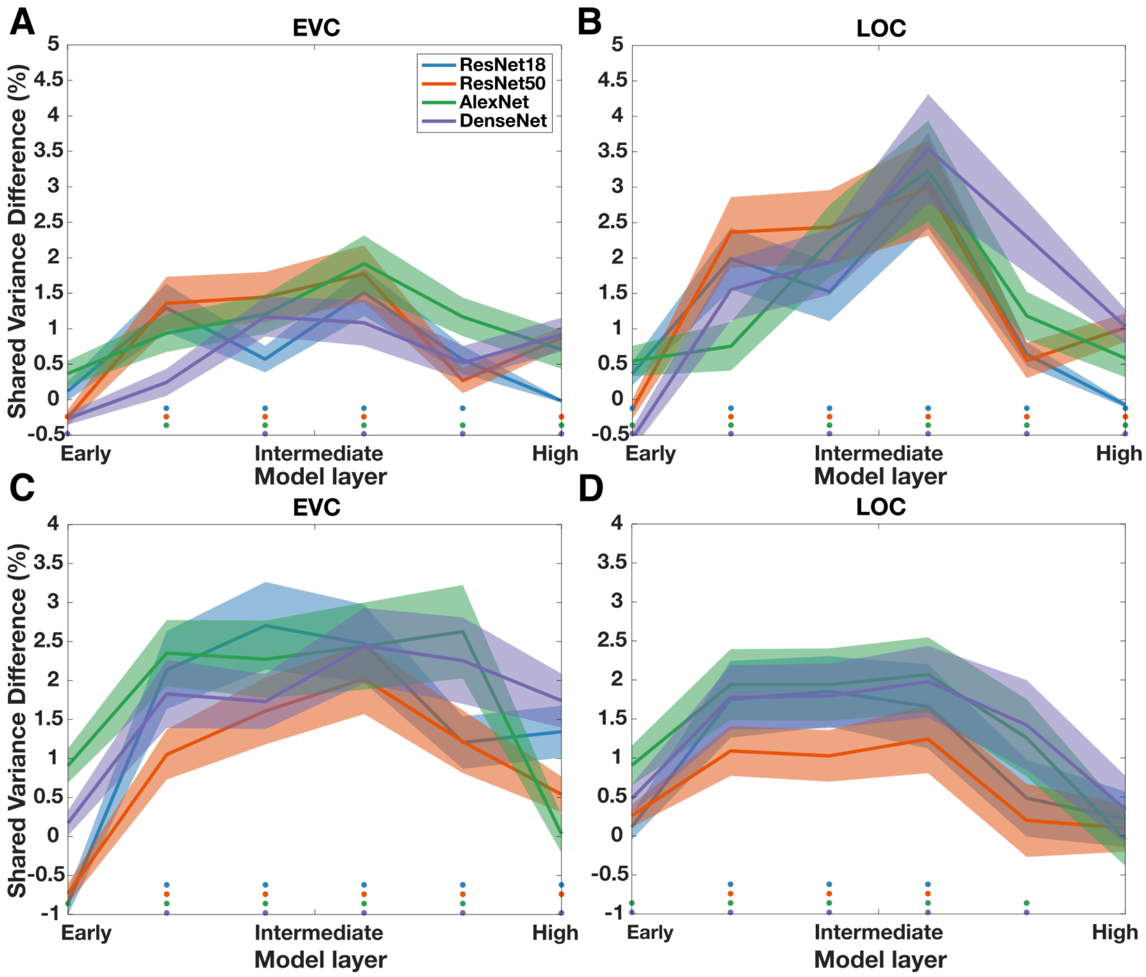

Supplementary Figure S3. Effects of network training on shared variance between networks, brain and behavior for man-made/natural and basic-level categorization tasks in EVC and LOC. A,B). man-made/natural categorization. Shared variance differences (%) between trained and untrained networks in early visual cortex (EVC, A) and lateral occipital cortex (LOC, B). Overall, most layers of trained networks showed significant increases in shared variance compared to the untrained networks. Only the first layer in DenseNet16 showed a decrease in shared variance. **C,D) Basic-level categorization: Shared variance differences for basic-level categorization in EVC (C) and LOC (D).** Similar to the results for man-made/natural categorization, there were significant increases in most layers in EVC. However, in LOC, effects were less widespread, with significant increases primarily in early to intermediate layers.

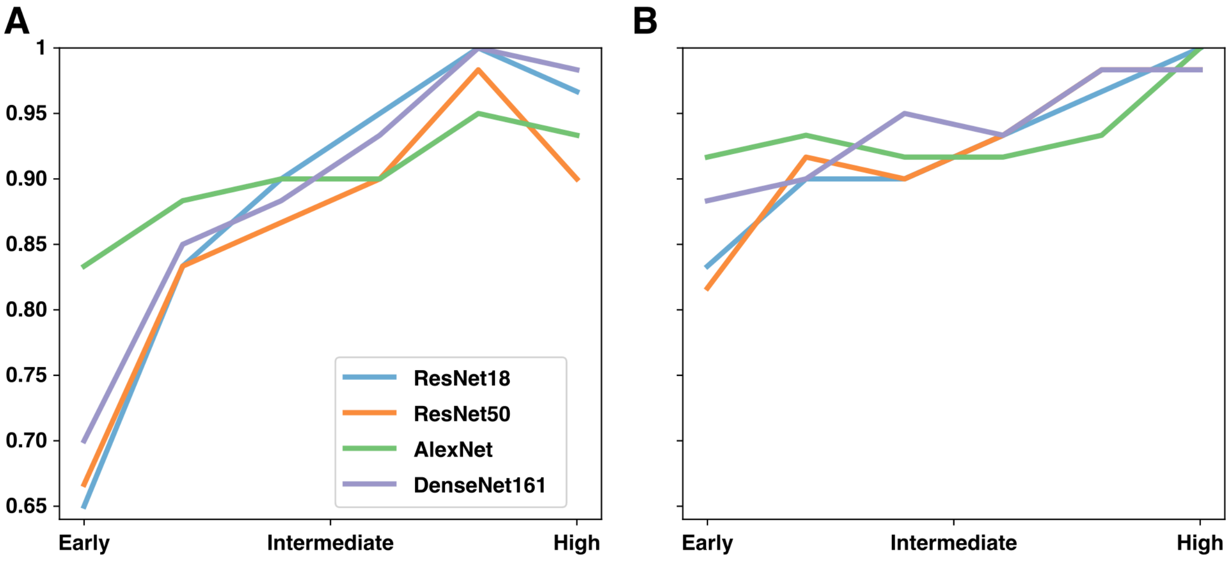

Supplementary Figure S4. Decoding accuracies based on network activations across different network architectures for man-made/natural categorization (A) and basic-level categorization (B). For both categorization tasks network accuracies increased gradually with layer depth. For man-made/natural categorization there was a notable drop in decoding accuracy in the last fully connected layer.
